# Taste Enhancement by Pulsatile Stimulation Is Receptor Based But Independent of Receptor Type

**DOI:** 10.1007/s12078-012-9126-8

**Published:** 2012-04-27

**Authors:** Kerstin Martha Mensien Burseg, Sara Marina Camacho, Johannes Hendrikus Franciscus Bult

**Affiliations:** 1 TI Food and Nutrition, P.O. Box 557, 6700 AN Wageningen, The Netherlands; 2 NIZO Food Research BV, P.O. Box 20, 6710 BA Ede, The Netherlands

**Keywords:** Pulsatile stimulation, Taste enhancement, Taste fusion period, Taste receptor, Gustometer

## Abstract

Effects of subjects’ taste sensitivity (expressed as taste detection threshold), tastant quality and taste transduction mechanism on pulsation-induced taste enhancement were tested. Taste intensities of pulsatile MSG and NaCl stimuli at pulsation periods below, at and above individual taste fusion periods (TFP in seconds) were compared to taste intensities of a continuous reference of the same net tastant concentration and quality. In line with results previously reported for sucrose, pulsation-induced taste enhancement peaked around TFP for both MSG and NaCl and did not require perception of tastant pulsation. TFP and pulsation effects were independent of the taste transduction mechanism (G-protein-coupled receptor for MSG versus ion-channel for NaCl). The absence of a relation between TFP and taste sensitivity suggests that temporal gustatory resolution and taste sensitivity are not necessarily influenced by the same factors. The results support earlier findings that early stages of taste transduction are involved in pulsation-induced taste enhancement. Pulsation-induced taste enhancement is determined by the pulsation rate (i.e. TFP) which is longer for MSG than NaCl. This is probably due to the tastant-specific interaction with the receptor rather than the taste transduction mechanism (G-protein-coupled receptor versus ion-channel) involved.

## Introduction

The successive alternation of high and low tastant concentrations (pulsatile tastant stimulation) results in higher taste intensity ratings than stimulation with the same net but non-alternating tastant concentration (Burseg et al. 2010a; Busch et al. 2009; Meiselman and Halpern 1973). This was demonstrated for pulsatile stimulation with sucrose (Burseg et al. 2010a, 2011a) and sodium chloride (Busch et al. 2009; Meiselman and Halpern 1973). As shown for pulsatile sucrose stimulation, the magnitude of pulsation-induced taste enhancement depends on the duration of the pulsation period, i.e. the summed durations of the high concentration tastant pulse and the low concentration interval (Burseg et al. 2010a). At sufficiently long pulsation periods, subjects perceive the fluctuation of taste intensities caused by the pulsation of tastant concentrations.

When pulsation rates increase (and the pulsation period decreases), taste fluctuation will become more difficult to distinguish, and consequently the probability that subjects detect taste fluctuations will decrease. At sufficiently high pulsation rates, subjects will no longer be able to distinguish between pulsatile and continuous taste stimuli on the basis of perceived taste intensity fluctuations alone. The pulsation period length at which there is a 50 % probability that a subject correctly discriminates between pulsatile and continuous taste stimulation (after correcting for the chance level of correct detections due to the used discrimination task) is the pulsation threshold. We refer to this pulsation threshold as the ‘taste fusion period’ (TFP) (Burseg et al. 2010a). We previously observed that sweet taste enhancement was highest at sucrose pulsation periods in the range of two times TFP_suc_ (subjects mostly detect tastant pulsation) to 0.5 times TFP_suc_ (subjects mostly fail to detect tastant pulsation) (Burseg et al. 2010a). We concluded that detection of the tastant concentration alternations upon pulsatile stimulation is no requirement for pulsation-induced sweet taste enhancement. The absence of a relation between the ability to perceive pulsation and the magnitude of pulsation-induced taste enhancement suggests that the enhancement stems from early stages of gustatory processing (Burseg et al. 2010a).

In humans, the temporal dynamics of neural gustatory responses depend on the taste-transduction mechanism involved (e.g. ion-channel activation for sodium- and proton-based stimulation, G-protein-coupled receptor activation for bitter and sweet taste stimulation (Breslin and Huang 2006)).This is, for instance, reflected in the observed variation in latencies between the onset of a taste stimulus and the onset of the corresponding primary cortical taste response (Kobayakawa et al. 2005; Saito et al. 1998). In line with this, gustatory reaction times (the minimum time required for a subject to report any taste change after onset of taste stimulation) in human adults also differ between taste qualities (Yamamoto and Kawamura 1981). We hypothesize that the ability of receptor cells to encode the changing tastant concentration of pulsatile stimuli at a given pulsation rate and as a function of time also depends on the taste transduction mechanism involved (e.g. G-protein-coupled receptor versus ion-channel). If so, both TFP as well as the size of pulsation-induced taste enhancement would then depend on the transduction mechanism involved. This was tested in the present study by comparing pulsation-induced taste enhancement for MSG and NaCl.

MSG receptors are members of class C G-protein-coupled receptors (GPCR) (Breslin and Huang 2006; Li et al. 2002; Lopez Cascales et al. 2010). Salt perception is mediated by a different mechanism involving epithelial sodium channels (e.g. ENaCs) (Stahler et al. 2008). Per subject, TFPs for MSG (TFP_MSG_) and NaCl (TFP_NaCl_) were determined at stimulus concentrations that produce equal perceived intensities. Taste intensities of pulsatile stimuli at individualized pulsation periods below, at and above TFP were then compared to a continuous reference of the same net tastant concentration and quality. This procedure allowed us to compare the effects of transduction mechanism (G-protein-coupled receptor versus ion-channel) on both TFP and the relative magnitude of pulsation-induced taste enhancement.

Apart from the reported taste pulsation period dependency of the invoked taste enhancement (Burseg et al. 2010a), there is further evidence that temporal aspects in stimulus presentation are linked with perceived taste intensity: (1) human gustatory reaction times decrease as the stimulus concentration increases (Yamamoto and Kawamura 1981; Yamamoto et al. 1985) and (2) human taste stimulus intensity increases with stimulus duration for stimuli that are sufficiently short to not cause adaptation of the gustatory system to presentations of that specific stimulus (Kelling and Halpern 1988).

The enhancement of taste intensities by temporal fluctuation of stimulus concentrations illustrates how temporal stimulus dynamics can alter the magnitude of the percept. The opposite, i.e. the magnitude of the stimulus concentration altering the temporal dynamics of the percept, is also observed as increasing the stimulus concentration lowers the gustatory reaction time in humans (Yamamoto and Kawamura 1981). This suggests that the stimulus concentration may also affect the TFP, with lower TFPs expected for higher stimulus concentrations. Given the fact that the perceived intensity of a taste solution depends on an individual’s sensitivity for that taste, TFP may vary between subjects as a function of their individual taste sensitivity. Therefore, we also tested the hypothesis that tastant-TFPs of individual subjects are related to their respective taste thresholds.

## Materials and methods

### Gustometer

An eight-channel computer controlled array of fluid pumps, which we will refer to as a gustometer (Bult et al. 2007), was operated as described earlier (Burseg et al. 2010a) to produce and deliver stimuli intra-orally with well-defined tastant concentrations that are varied over time and at a total flow rate of 15 mL/min. Subjects held a mouthpiece between the incisors that protruded approximately 10 mm into the mouth and that presented stimuli directly on the distal portion of the dorsal anterior tongue. Subjects swallowed at will.

### Subjects

Sixteen paid subjects (age 30–58 years, three male) were recruited. All had participated earlier in gustometer studies. As reports indicate that a part of the population is unable to distinguish MSG from NaCl, e.g. are unable to detect the umami taste component in addition to the salty component of MSG (Lugaz et al. 2002), subjects were pre-screened for their ability to distinguish between MSG and NaCl stimuli (20 mM each). Subjects participated if they correctly identified the odd stimulus in at least seven out of ten triangles of MSG and NaCl combinations. This selection criterion corresponds to a 2.0 % chance probability of identifying MSG-non-tasters as tasters according to the binomial distribution for *p* = 1/3 (triangle method). Subjects were experienced users of the labeled magnitude scales (Green et al. 1996) and received a 1-h training session where they were instructed to link verbal references with MSG and NaCl reference solutions at varying concentrations. Subjects were instructed to consume only water 1 h prior to the test. Materials and methods used did not require medical ethical approval under Dutch regulations (food grade ingredients, oral delivery). Moreover, the study was performed according to an internal standard procedure that was designed for studies involving human volunteers. Subjects gave written informed consent.

### Determination of iso-intense MSG and NaCl concentrations

#### Stimuli

Aqueous (Evian, Danone, France; conductivity 560 mS/cm) solutions of food grade MSG (Ajinomoto Foods Europe SAS, Germany; purity > 99.0 %) or food grade NaCl (‘suprasel extra fine’, CA FNZ industrial B.V., The Netherlands; purity > 99.9 %) were presented at predefined ratios by a gustometer. Concentration series for MSG and NaCl ranged from 2.0 to 89 mM (MSG; 12 steps) and 6.0–253 mM (NaCl; 12 steps). Starting from 2.0 mM (MSG) and 6.0 mM (NaCl), concentrations were systematically increased by a multiplication factor of 1.412 to obtain approximately equal perceptual distances in taste intensities between stimuli of successive tastant concentrations.

#### Method

Stimuli were delivered intra-orally over 20 s. Taste intensity ratings for MSG and NaCl were rated on labeled magnitude scales (Green et al. 1996) that were displayed on paper. Determination of iso-intense MSG and NaCl concentrations was repeated over two independent sessions.

#### Data analysis

Individual psychophysical taste curves were obtained for NaCl and MSG by plotting the logarithms of average taste intensity ratings over stimulus repetitions as a function of log-concentrations (in millimolar). Linear functions were fit to the psychophysical taste curves and used to estimate individual intensity ratings for the 20-mM MSG stimulus. These intensities were used to estimate individual NaCl concentrations, equi-intense to the 20-mM MSG stimulus. This NaCl concentration was then used for determination of the individual TFP NaCl (see the next section).

### Determination of TFP

#### Stimuli

Taste solutions pulsed at variable pulsation periods (MSG: 20 mM; NaCl: individually defined concentrations iso-intense to 20 mM MSG) and interleaved with water intervals (Evian, Danone, France; conductivity 560 mS/cm) were delivered by the gustometer as described previously in the article. Subjects swallowed stimuli at will. After every 10th sample, a break of 5 min was given. Between samples, subjects rinsed their mouth with water.

#### Method

The TFP is defined as ‘the threshold of pulsation perception’. By definition, this is the pulsation period duration at which subjects correctly identify pulsed stimuli in 50 % of the cases (corrected for chance), in comparison to continuous stimuli. When using a binary identification method (‘2-AFC’ or ‘A–not A’, this would correspond to 75 % correct identifications of a pulsed stimulus at a given pulsation period duration and after a fair amount of comparisons (as would be the case if the method of constant stimuli were used to assess the threshold). In the present study, individual TFPs were assessed using the less time-consuming method of limits, also referred to as the staircase method. In particular, we used an A–not A procedure presenting pulsed taste stimuli at variable pulsation rates. Subjects started with a stimulus pulsed with an 8-s pulsation period. Up-down rules were defined as follows: after two consecutive correct pulsation identifications, the pulsation period was lowered with 0.25 s. After one failed pulsation identification, the pulsation period was raised with 0.25 s. Pulse and interval durations were always the same (e.g. an 8-s pulsation period consisted of a 4-s pulse and a 4-s interval) to obtain a pulse–interval duration ratio of 1.

The threshold estimate obtained with a staircase method depends on the up-down rule that is applied (see for instance Brown 1996). If the number of staircase reversals approaches infinity, the observed threshold for a given pulsation period duration will converge to the period duration for which the probability of an upward reversal equals the probability of a downward reversal. Given a true detection probability *p* of a pulsed stimulus, and given the generic rule that *n* consecutive correct identifications provoke a downward step in period duration and any missed pulsed stimulus in a sequence of *n* stimuli to provoke an upward step, the staircase converges to the period duration for which the probability of producing *n* correct identifications (*p* ^*n*^) equals the probability of *not* producing *n* consecutive correct identifications (1 − *p* ^*n*^), or *p* ^*n*^ = 1 − *p* ^*n*^. Resolving for *n* = 2, this predicts that the used two-down one-up rule converges at a pulsation frequency for which *p* = (0.5)^1/2^ = 0.71. Hence, the two-down one-up rule produces a slightly underestimated TFP.

#### Data analysis

Individual TFPs were calculated as geometric mean of the last four staircase reversal points of a total of seven reversals.

### Determination of the taste detection threshold

#### Stimuli

The MSG or NaCl stimuli were delivered by running two pumps in parallel, mixing MSG or NaCl solutions with water (Evian, Danone, France; conductivity 560 mS/cm) at predefined ratios. The MSG concentration series was defined as successive increments or decrements of concentrations from 5.9 mM/L by a factor of 1.412. The concentration series of NaCl was defined as increments or decrements of concentrations from 18.4 mM/L with the same multiplication factor of 1.412.

#### Method

Subjects were presented with two stimuli, one of them being water only while the other stimulus containing water with the tastant. The MSG starting concentration was 5.9 mM/L. The NaCl starting concentration was 18.4 mM/L. Stimuli were presented at a flow rate of 15 mL/min during 20 s each with a 3-s break between stimuli. Following a staircase procedure, subjects were instructed to indicate the solution containing the tastant according to the 2-AFC method (Burseg et al. 2010a). Concentrations were lowered by one step if two consecutive tastant solutions were identified correctly. If water was identified as the tastant solution in the first or second comparison, the tastant concentration was raised by one step (one-up, two-down rule). Detection thresholds for MSG and NaCl were determined over two separate sessions. The order of sessions was balanced over subjects. Between stimuli, at least 1 min was given to rinse the mouth with water. After every 5th comparison, subjects were given a 5-min break. At the beginning of each session, subjects received two warm-up stimuli.

#### Data analysis

Stimulus thresholds concentrations (in millimolar per liter) were calculated as the geometric means of the last four concentration reversals.

### Pulsation-induced enhancement for MSG and NaCl

#### Stimuli

High intensity pulses (MSG: 20 mM; NaCl at the individual’s iso-intense NaCl concentration) and water intervals were delivered by the gustometer as described previously in the article. Subjects were presented with stimuli of individualized pulsation periods depending on their TFPs, defined as of 2, 1 and 0.5 × TFP for MSG stimuli and 4, 2, 1, 0.5 and 0.25 × TFP for NaCl stimuli. Stimulus pulsation was continued (4 × TFP: one time; 2 × TFP: two times; 1 × TFP: four times; 0.5 × TFP: eight times; 0.25 × TFP: 16 times) to yield stimuli of equal duration. The pulse/interval ratio was kept at 1 to yield an average MSG concentration of 10 mM. The average NaCl concentration depended on the individual’s pulse concentration that was iso-intense with 20 mM MSG. For example, if a subject rated 20 mM MSG and 25 mM NaCl as iso-intense, the NaCl pulse concentration was 25 mM and the average period concentration was 12.5 mM. In addition, a continuous reference of the same duration and same net concentration (MSG: 10 mM; NaCl: average NaCl concentration depended on the individual’s pulse concentration that was iso-intense with 20 mM MSG; see above) was given. This yielded a total of four different MSG stimuli and six different NaCl stimuli.

Stimulus concentrations were verified by potentiometric determination of MSG or NaCl concentrations collected over 1 min (Conductivity Meter Radiometer, CDM 92, Electrode CDC 64 IT, Radiometer, Copenhagen). The electrode was calibrated with MSG or NaCl solutions of known concentrations.

#### Method

During sample presentation, subjects rated taste intensity over time (time–intensity; scale 0–100; anchored ‘not intense’–‘very intense’) by moving the control of a vertical rating-bar (100 mm) on the computer screen by manipulating a computer mouse. The evaluation included intensity ratings upon stimulation and continued after stimulus termination to allow subjects to report after-taste intensities. Subjects evaluated each MSG stimulus six times (*n* = 24) and each NaCl stimulus four times (*n* = 24) in four separate sessions. In each session, only one taste quality was evaluated (MSG or NaCl). The order of tastants presented per session was randomized over the sessions. In one session, 12 stimuli were presented in three groups of four stimuli each, separated by 5-min breaks. Each group was preceded by a continuous reference (MSG or NaCl respectively) for self-calibration. The continuous references were also included as blind sample. Subjects paused at least 1 min between stimuli to rinse the mouth with water. At the beginning of each session, subjects received two additional warm-up stimuli.

#### Data analysis

The area under the curve (AUC) of taste intensity over time (e.g. Burseg et al. 2010b, 2011b) served as a measure for MSG and NaCl taste intensity. After-taste intensity recordings were excluded from AUC calculation. Main effects of pulsation period (fixed factor; MSG: four categories [2, 1 and 0.5 × TFP, continuous reference]; NaCl: six categories [4, 2, 1, 0.5 and 0.25 × TFP, continuous reference]) on AUC were calculated by one-way ANOVA. Post-hoc comparisons were made using Tukey’s HSD corrections for multiple comparisons. All tests were at a significance level of *α* = 0.05.

In a subsequent ANOVA, effects on AUC of pulsation period (fixed factor; MSG: four categories [2, 1 and 0.5 × TFP, continuous reference]; NaCl: six categories [4, 2, 1, 0.5 and 0.25 × TFP, continuous reference]), replicate (fixed factor; MSG: six replicates; NaCl: four replicates) and subjects (random factor) were tested by univariate ANOVA. The tests included main effects and all two-way interactions (SPSS, Chicago, version 17).

As a measure of lingering of after-taste, half times (*t*_0.5_) were calculated for intensity ratings produced after the moment of stimulus termination. For this, the exponential decay function *y* = *Ae* ^−*λt*^, with *A* = the taste intensity at *t* = 0 (moment of stimulus termination) and *λ* = decay rate (per second), was fitted to individual after-taste curves. Model fits were optimized by the iterative ‘fminsearch’ algorithm in Matlab (version 7.0.1; the Mathworks Inc, Natick, MA), minimizing the sum of squared differences between observed and modeled intensity ratings. Parametric correlation coefficients, *R*, for observed intensities and their respective modeled intensities were calculated as measures of individual function-fit qualities. Decay rates that stemmed from curve fits with *R* equal to or higher than 0.8 were used to calculate half times $\left( {{t_{0.5}} = \frac{{{ \ln }2}}{\lambda }} \right)$ of after-taste for single taste intensity rating curves. To accommodate for similarity of functions with very large positive or negative half times (both of which produce near-to-flat decay functions), inverse half times (*t*_0.5_))^−1^ were used as dependent variable for the statistical *t*-test (SPSS, Chicago, version 17) comparing the effects of tastant (MSG versus NaCl) on half times.

## Results

### Determination of iso-intense MSG and NaCl concentrations

Iso-intense NaCl concentrations for 20-mM MSG reference solution ranged from 23 to 90 mM (median 64 mM).

### Determination of TFP

**Fig. 1:**
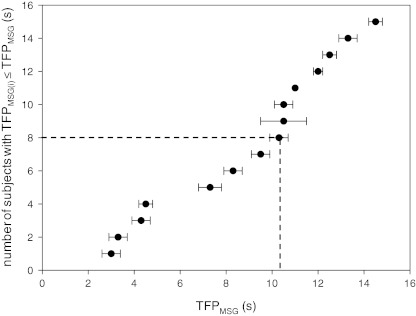
Number of subjects out of 15 with individual MSG taste fusion periods (TFP_MSG(i)_ in seconds) equal to or smaller than the MSG taste fusion period (TFP_MSG_ in seconds). *Dashed line* represents median TFP = 10.3 s. Error bars represent standard error of four repetitions

**Fig. 2:**
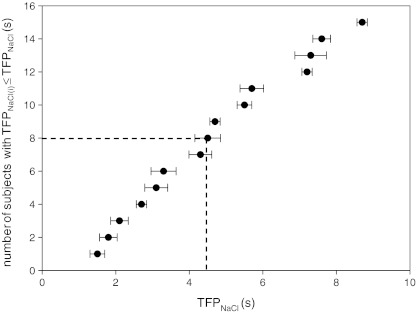
Number of subjects out of 15 with individual NaCl taste fusion periods (TFP_NaCl(i)_ in seconds) equal to or smaller than the NaCl taste fusion period (TFP_NaCl_ in seconds). *Dashed line* represents median TFP = 4.4 s. Error bars represent standard error of four repetitions

The median TFP_MSG_ (10.3 s; range 3.0–14.5 s; *Δ* = 11.5 s; Fig. 1) was more than two times larger than the median TFP_NaCl_ (4.4 s; range 1.5–8.7 s; *Δ* = 7.2 s; Fig. 2).

### Determination of taste detection thresholds

Taste detection thresholds differed between subjects and taste modalities. The median MSG threshold was 0.87 mM/L (0.30–1.14 mM/L), and the median NaCl threshold was 5.29 mM/L (range 2.84–8.29 mM/L).

### Pulsation-induced taste enhancement for MSG and NaCl

**Fig. 3:**
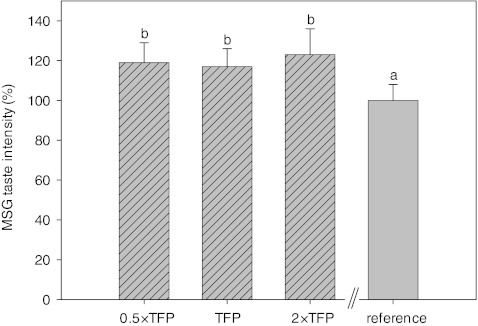
Relative MSG taste intensities of pulsatile stimuli at pulsation periods below the taste fusion period (TFP; 0.5 × TFP_MSG_), at TFP and above TFP (2 × TFP) compared to continuous MSG delivery of the same net MSG concentration (reference; 100 %). *Dashed bars* indicate pulsatile stimuli. Error bars express standard error (*n* = 6 repetitions). Samples denoted with the same *letter* are not significantly different at *p* < 0.05

**Fig. 4:**
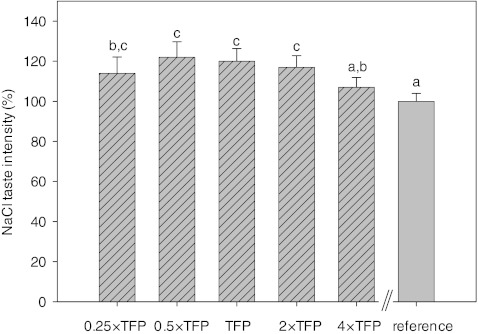
Relative NaCl taste intensities of pulsatile stimuli at pulsation periods below the taste fusion period (TFP; 0.25 and 0.5 × TFP_NaCl_), at TFP and above TFP (2 and 4 × TFP) compared to continuous NaCl delivery of the same net NaCl concentration (reference; 100 %). *Dashed bars* indicate pulsatile stimuli. Error bars express standard error (*n* = 4 repetitions). Samples denoted with the same *letter* are not significantly different at *p* < 0.05

One-way ANOVA showed that taste intensities differed significantly between pulsation conditions for MSG ([*F*(3,8791) = 6.29; *p* < 0.001]) and NaCl ([*F*(5,6897) = 10.84; *p* < 0.001]). Despite equal net tastant concentrations, pulsatile stimuli were rated about 20 % more intense than the continuous references for MSG (Fig. 3) and NaCl (Fig. 4). Post-hoc analysis revealed that only pulsed NaCl stimuli at pulsation periods 4 × TFP were not rated as more intense than the continuous reference. Instead, 4 × TFP stimuli produced significantly lower intensity ratings than the other pulsatile stimuli (Fig. 4). For both tastants, univariate ANOVA revealed significant intensity effects by stimulus (MSG: [*F*(3, 39) = 4.79; *p* < 0.01]; NaCl: [*F*(5, 75) = 5.51; *p* < 0.001]) and subject (MSG: [*F*(13, 60.1) = 3.26; *p* < 0.01]; NaCl: [*F*(15, 80.25) = 8.26; *p* < 0.001]), and significant stimulus by subject interactions (MSG: [*F*(39, 195) = 2.56; *p* < 0.001]; NaCl: [*F*(75, 375) = 6.16; *p* < 0.001]) and replicate by subject interactions (MSG: [*F*(65, 195) = 2.41; *p* < 0.001]; NaCl: [*F*(75, 375) = 1.39; *p* < 0.05]).

Inverse half times of after-taste functions differed significantly between MSG and NaCl [*t*(410.2) = 2.204, *p* = 0.028]. The corresponding panel half-life time of MSG was 5.9 s, while the panel half-life time of NaCl was 3.5 s.

## Discussion

In line with previous studies, pulsatile stimuli were rated as more intense than a continuous reference of the same net tastant concentration. Both MSG and NaCl stimuli were rated 20 % more intense than the continuous reference if presented at pulsation periods 0.5–2 × TFP. As shown for NaCl, outside this range (e.g. at 4 × TFP), pulsatile stimuli were no longer rated more intense than the continuous reference. Moreover, taste enhancement did not depend on the subject’s ability to perceive pulsed taste: equal taste intensity ratings were obtained for stimuli given at pulsation periods two times above (pulsation is perceived) and two times below TFP (pulsation is not perceived). This is in line with earlier observations made for sweet taste enhancement (Burseg et al. 2010a). The repeatability of these findings, i.e. stable enhancement around TFP and independence of ability to perceive pulsation at a given pulsation period, over different taste qualities (sweet, umami and salty taste) and taste transduction mechanisms (G-protein versus ion-channel) suggests that pulsation-induced taste enhancement is an inherent property of the human taste system rather than an isolated phenomenon observed for a specific tastant/taste transduction mechanism.

It has been suggested earlier that pulsation-induced taste enhancement is reflected in different chorda tympani response patterns for continuous and pulsatile stimuli (Meiselman and Halpern 1973). Upon continuous stimulation, chorda tympani responses are characterized by an initial high frequency (phasic response) that decreases rapidly to a lower steady state level. Upon intermittent taste presentation, however, the rat chorda tympani shows a rhythmic, serial phasic burst pattern (Halpern and Marowitz 1973). This entails higher total burst counts than continuous stimuli at the same net concentrations produce. Similarly, in humans, discontinuous, pulsatile tastant stimulation may induce such a serial phasic response pattern. The overall higher neural output would then explain taste intensity enhancement for pulsatile stimuli (Meiselman and Halpern 1973). Such a mechanism based on serial phasic response patterns at post-receptor levels still accounts for the present results since it predicts enhancement independent of the taste transduction mechanism involved.

An important factor modulating the magnitude of pulsation-induced taste enhancement is the pulsation period. In line with the observations made for alternating sucrose solutions (Burseg et al. 2010a), taste intensity peaked around TFP (0.5–2 × TFP). In the present study, NaCl intensity enhancement disappeared at low pulsation rates when as pulsation was clearly perceivable (4 × TFP). This may indicate that at larger period durations, subjects averaged taste intensities of perceived pulsatile stimuli over pulsation periods, as suggested by the fact that the total area under the taste curve leveled off to the taste intensity of the continuous reference. This seems plausible as the concentration of the continuous reference was equal to the averaged period tastant concentration. On the other hand, the apparent pulsation period dependency suggests a strict temporal window (e.g. close to TFP) to evoke the effect. A similar phenomenon was described for vision (Macknick 2006; Wu et al. 1996) by the observation that a flickering light is perceived as brighter compared to a steady light of the same average luminance (Brücke–Bartley effect (Wu et al. 1996)). Brightness enhancement was partly related to the occurrence of serial phasic response patterns of the optic nerve and subsequent larger accumulated fiber responses. These response patterns are restricted to flicker frequencies around the so called ‘flicker fusion point (FFP)’, where a flickering stimulus fuses into a continuous percept. The similarity between brightness enhancement at flicker fusion and taste enhancement around TFP then supports the above-mentioned theory of the occurrence of serial phasic receptor responses of chorda tympani upon pulsatile stimulation. This needs to be confirmed in electrophysiological studies.

McBurney studied taste sensitivity to stimuli that vary in tastant concentration as a function of time (McBurney 1976). Subjects’ ability to detect tastant concentration fluctuations increased with the size of concentration difference between alternating stimuli (expressed as percent modulation at threshold), and, similar to the present study, the frequencies the taste stimuli were alternated at. Threshold–frequency functions varied across taste qualities with greatest sensitivity for salty and sweet stimuli. McBurney related the order of sensitivity found to the ‘order of reaction time and time of buildup of sensation to rectangular onset of taste stimuli’ as described earlier (Bujas 1935). This theory may equally account for TFP as it expresses the threshold period duration for taste pulsation perception and, like the gustatory reaction time, can be regarded as a measure for gustatory temporal resolution.

TFP_MSG_ (10.3 s) was approximately twice the magnitude of TFP_suc_ and TFP_NaCl_. The difference between TFP_suc_ and TFP_MSG_ cannot be attributed to the underlying taste transduction mechanism. The difference between TFP_MSG_ and TFP_NaCl_ cannot be attributed to intensity differences as MSG and NaCl intensities were matched. One explanation for the difference between TFP_MSG_ and TFP_NaCl_ is offered by the distinct MSG taste intensity profile that is characterized by a persistent after-taste (Giovanni and Guinard 2001). Sucrose (Pfeiffer et al. 2000) and NaCl (O’Mahony and Wong 1989), in contrast, show a more rapid decline in taste intensity after stimulation termination. This was also observed in the present study as indicated by a larger MSG intensity decay coefficient compared to NaCl. The molecular basis for the MSG after-taste is not known. The temporal profile of a taste sensation is not only shaped by the rate at which the tastant binds to and activates the receptor. The rate at which the tastant leaves the receptor is furthermore of importance (Beidler 1954; Pfeiffer et al. 2000). MSG after-taste may therefore be explained with a lower desorption rate compared to, for example, the non-lingering sucrose. Moreover, it has been suggested that certain (amphipatic) tastants (e.g. sweet and bitter compounds) not only interact with the outer membrane of the taste receptor but may migrate into the taste cell where they interact with downstream components that are responsible for the delay of signal termination of the taste sensation (Zubare-Samuelov et al. 2005). A similar mechanism may apply for MSG and explain the after-taste. We therefore propose that TFP is determined by the tastant-specific interaction with the receptor (including adsorption and desorption) rather than the taste transduction mechanism.

In line with TFP_suc_ (Burseg et al. 2010a), the TFP range measured for both TFP_MSG_ and TFP_NaCl_ suggests considerable between-subject variations and, subsequently, between-subject differences in gustatory temporal resolution. Subjects also vary noticeably in taste sensitivity (Krueger et al. 2006; Lim et al. 2008; Mojet et al. 2005; Shigemura et al. 2006). Given the relationship between taste sensitivity and temporal aspects in tastant presentations (e.g. as expressed by increase in taste intensity with stimulus duration (Kelling and Halpern 1988)), we assumed a relationship between TFP and taste detection threshold. Interestingly, this was not confirmed in the present study for both MSG and NaCl. This suggests that factors determining gustatory temporal resolution (i.e. TFP) do not necessarily account for differences in taste sensitivity. Alternatively, as subjects swallowed at will and tongue movements were not constraint, the individual differences in TFP may be explained by between-subject variations in intra-oral processing of (pulsatile) taste stimuli. Despite possible between-subject variations in oral processing, subjects seem to apply a consistent evaluation technique across stimuli as shown by the calculated standard errors upon TFP determination (Figs. 1 and 2; intra-subject variation). To further investigate this theory, a taste delivery system that eliminates possible individual differences in intra-oral shown stimulus processing as described elsewhere (Meiselman and Halpern 1973) could be applied.

Regarding the observed TFP variation over subjects, it should be noted that the used method for the assessment of TFP- and taste-thresholds is sensitive for the relative tendency of subjects to produce affirmative responses in the used detection task. In signal-detection theory, this tendency is referred to the ‘response criterion’ (Swets 1961). The choice to only present pulsed stimuli at various frequencies in the TFP assessment rules out the quantification of the response criterion. However, the consistent staircase reversals suggest that no panelists adopted extremely high response criteria, since that would have produced erratic stimulus identifications as a function of pulsation frequency. In addition, a general tendency to adopt high response criteria by some subjects would have produced a high correspondence between taste detection thresholds and TFP, which was not observed.

## Conclusions

The present study on pulsatile stimulation of MSG and NaCl replicated results previously reported for sucrose: (1) pulsation-induced taste enhancement peaks around TFP and (2) perception of pulsation is no requirement for enhancement. TFP and pulsation effects were independent of the taste transduction mechanism (G-protein receptor versus ion-channel), but TFP seems to be determined by the tastant-specific interaction with the receptor. The absence of a relationship between TFP and taste detection threshold suggests that the temporal resolution of taste perception and taste sensitivity are not necessarily influenced by the same factors. Overall, the results support the theory of involvement of early stages in taste processing in pulsation-induced taste enhancement. Pulsation-induced taste enhancement is determined by the pulsation rate (i.e. TFP). Besides the pulsation rate dependency, there seems to be no further dependency of enhancement on taste transduction mechanisms as such.
